# Immediate Implant Placement with Immediate Restorations after Dental Avulsions Due to Endotracheal Intubation in a Patient with Severe Chronic Periodontal Disease

**DOI:** 10.3390/diagnostics14090966

**Published:** 2024-05-06

**Authors:** Alexandre Perez, Adriana Fernandez Bargiela, Tommaso Lombardi

**Affiliations:** 1 Unit of Oral Surgery and Implantology, Division of Oral and Maxillofacial Surgery, Department of Surgery, Geneva University Hospitals, Faculty of Medicine, University of Geneva, 1205 Geneva, Switzerland; adriana.fernandezbargiela@hcuge.ch; 2 Unit of Oral Medicine and Oral Maxillofacial Pathology, Division of Oral and Maxillofacial Surgery, Department of Surgery, Geneva University Hospitals, Faculty of Medicine, University of Geneva, 1205 Geneva, Switzerland; tommaso.lombardi@unige.ch

**Keywords:** dental implant, immediate implant, immediate restoration, periodontitis, trauma, endotracheal intubation

## Abstract

We report the case of a 61-year-old woman who was referred to the Oral Surgery and Implantology Unit of the University Hospitals of Geneva to take care of edentulous sites after the dental avulsions of teeth 11 and 21 following traumatic shock due to endotracheal intubation under general anesthesia. The dental history revealed that the patient had a history of generalized chronic periodontitis that had been treated several years earlier. The treatment consisted, for the first time to our knowledge, of the immediate insertion of implants at sites 11 and 21 with simultaneous bone augmentation in a single surgical procedure and immediate restorations.

Periodontitis, the major cause of tooth loss in adults [1], is an infection-driven inflammatory disease in which the composition of the dental plaque, a structurally and functionally organized biofilm, plays a significant role. Dental plaque accumulation at the gingival margin initiates an inflammatory response that, in turn, may lead in susceptible individuals to the irreversible destruction of the periodontal attachment and alveolar bone. This pathological condition represents in addition a risk factor in implant therapy and constitutes a challenge for fixed reconstructions on implants and even more in the context of immediate implant placement [2,3]. Teeth avulsion is one of the most common dental injuries during general anesthesia associated with laryngoscopy for patients with advanced periodontitis [4] that can be detrimental to the patient’s well-being, especially when the patient should not expect complications which significantly disrupt normal function and the quality of life [5,6].

A 61-year-old woman was referred to the Oral Surgery and Implantology Unit of the University Hospitals of Geneva for the treatment of edentulous sites after the dental avulsions of teeth 11 and 21 following traumatic shock due to endotracheal intubation under general anesthesia performed to remove a laryngeal cyst. Past medical history was significant for pharyngeal dysesthesia and a supraglottic cyst. The dental history revealed that the patient had a history of chronic generalized periodontitis treated a few years earlier. Clinical and radiological examination showed generalized bone loss, especially at the edentulous sockets 11 and 21 and adjacent teeth 12 and 22 (Figure 1).

The treatment plan was based on periodontal maintenance and the replacement of edentulous sites 11, 21 using two single crowns screwed onto implants. Information regarding implant timing and therapeutic options for temporary prostheses was given to the patient, including the benefits and risks. The timing of the implantation chosen and accepted by the patient was immediate implant placement and a provisional prosthetic phase based on two immediate restorations using temporary crowns screwed in resin under occlusion.

Our treatment consisted, for the first time to our knowledge, upon the patient’s awakening from general anesthesia and with her consent, of the replacement of the avulsed teeth by immediately inserting dental implants followed by immediate screw-retained coronal restorations (Figure 2). The immediate placement of dental implants in extraction sockets has proven to be a safe and viable treatment option in the case of socket type I [7]. This procedure became a strategy favored by clinicians owing to the associated advantages, such as reducing the number of surgical procedures, stress on the patient and morbidities, shortening treatment duration, and a better management of soft tissue and alveolar morphology. In our case, the socket was not ideal because it had suffered bone loss as part of the history of periodontal disease. Several studies have also reported that the high success rates of this technique were associated with the achievement of primary stability [8]. Bone anchorage for primary stability in immediate implantation primarily depends on the bone at the apical and palatal levels of the alveolus [8]. For this reason, particular attention must be paid to the proximity of neighboring anatomical structures such as the maxillary sinuses, nasal cavities, mandibular canal, and mental foramina. In the case of post-extraction sockets in the context of a reduced periodontium, achieving primary stability can be even more complex due to the reduced amount of bone and requires a more meticulous and precise surgical procedure. In our patient, one of the difficulties was to obtain primary stability because the residual bone height between the apical level of the sockets and the nasal cavity was 6 mm, which greatly limited the possibility of apical anchorage. Typically, bone augmentation simultaneous with immediate implantation is required to fill the gaps between the vestibular cortex and the exposed implant surface [8]. In our case, after placing the implants, the filling was carried out. However, the socket being of reduced height, the part of the dehiscent implant surface was limited to only 3 mm. Immediate implantation also offers the possibility of an immediate fixed provisional restoration, provided that primary stability is achieved and a sufficient insertion torque of around 35 N/cm is applied [9]. In our patient, although the periodontium was reduced in the context of a history of generalized chronic periodontitis, an insertion torque of 38 and 40 N/cm was obtained at the level of implants 11 and 21, respectively, and immediate restorations were carried out for the first time in this context. The patient followed regular dental and dental prosthesis checkups as well as oral hygiene recall appointments three times a year. During these appointments, the examiner recorded the clinical periodontal parameters and checked the status of the prosthesis. At the end of the appointments, a session of prophylaxis was performed, as necessary. At 3-year follow-up, she showed functional results without any recurrence or complications observed and the patient was fully satisfied with the treatment received (Figure 3). Our case highlights that immediate implant placement with immediate restoration can be a good valid alternative to standard treatment in case of a non-ideal socket with a history of periodontitis.

## Figures and Tables

**Figure 1:**
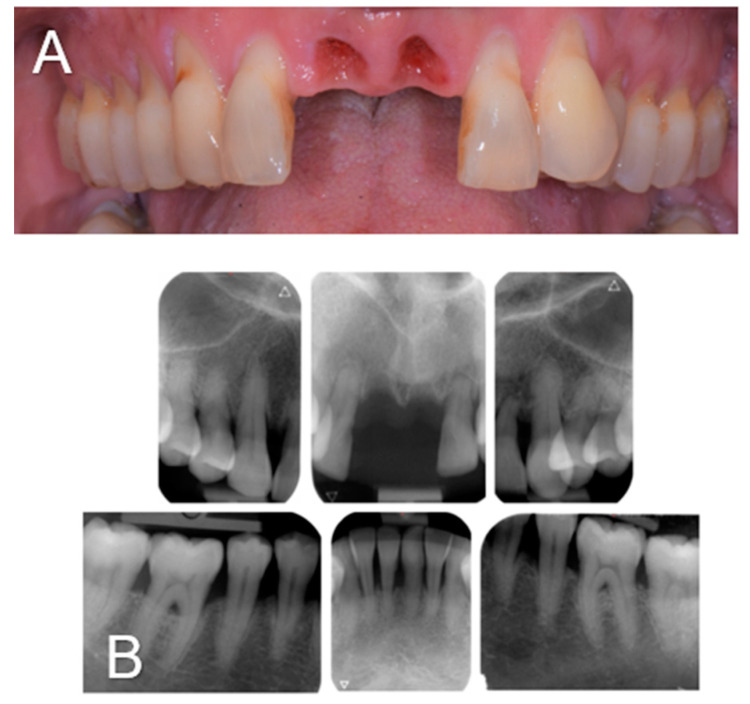
Clinical view (**A**) and intra-oral radiographs (**B**) showing the initial clinical status after dental avulsions due to endotracheal intubation as well as the severe chronic periodontal disease.

**Figure 2:**
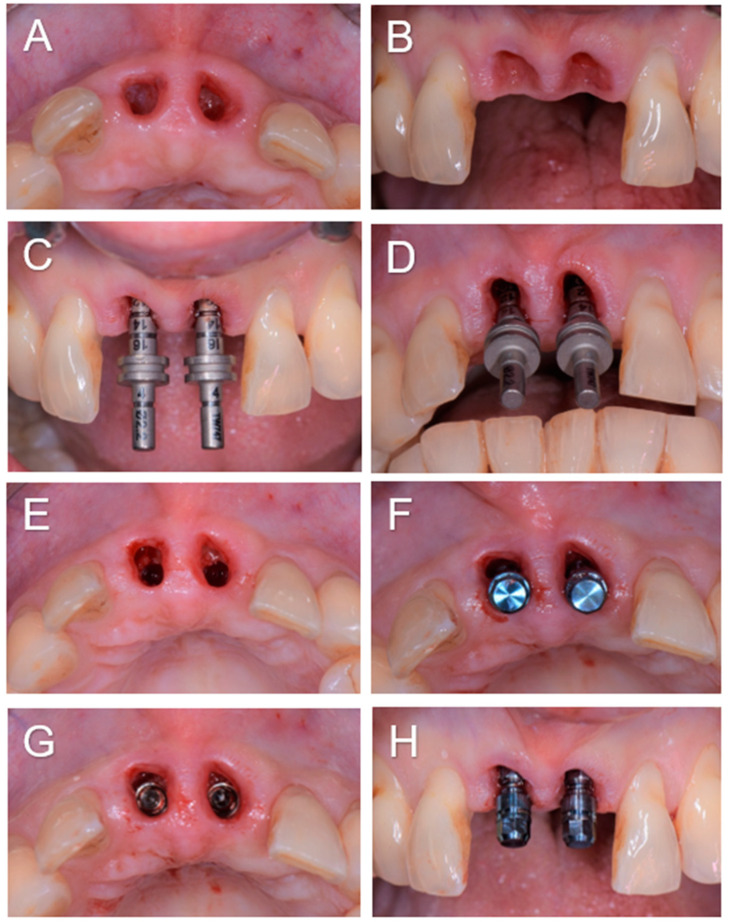

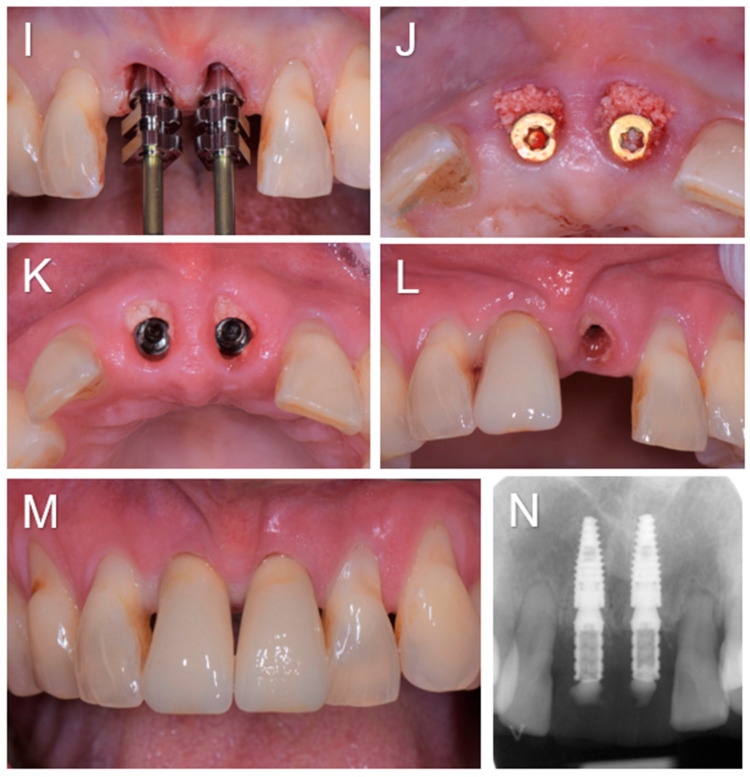
(**A**–**N**). Intraoperative view showing sockets after curettage (**A**,**B**) and the control of the drilling axes of sites 11 and 21 (**C**,**D**), and an occlusal view showing implant bed preparation (**E**). The occlusal (**F**,**G**) and buccal (**H**) view after the placement of implants (Bone Level, Ø3.4 × 10 mm, Straumann, Switzerland) at sites 11 and 21, showing small buccal bone defect ‘’dehiscence-like’’ at sites 11 and 21. The buccal view with transfer device screwed onto the implants for impression taking (**I**). The clinical view showing the augmented sites using autogenous bone chips and alloplastic bone (BoneCeramic™, Straumann, Switzerland), after the impression (**J**) and 3 h later (**K**) during the fixation of the screw-retained temporary crowns (**L**,**M**) and the periapical 2D radiographs taken immediately after implant placement (**N**).

**Figure 3:**
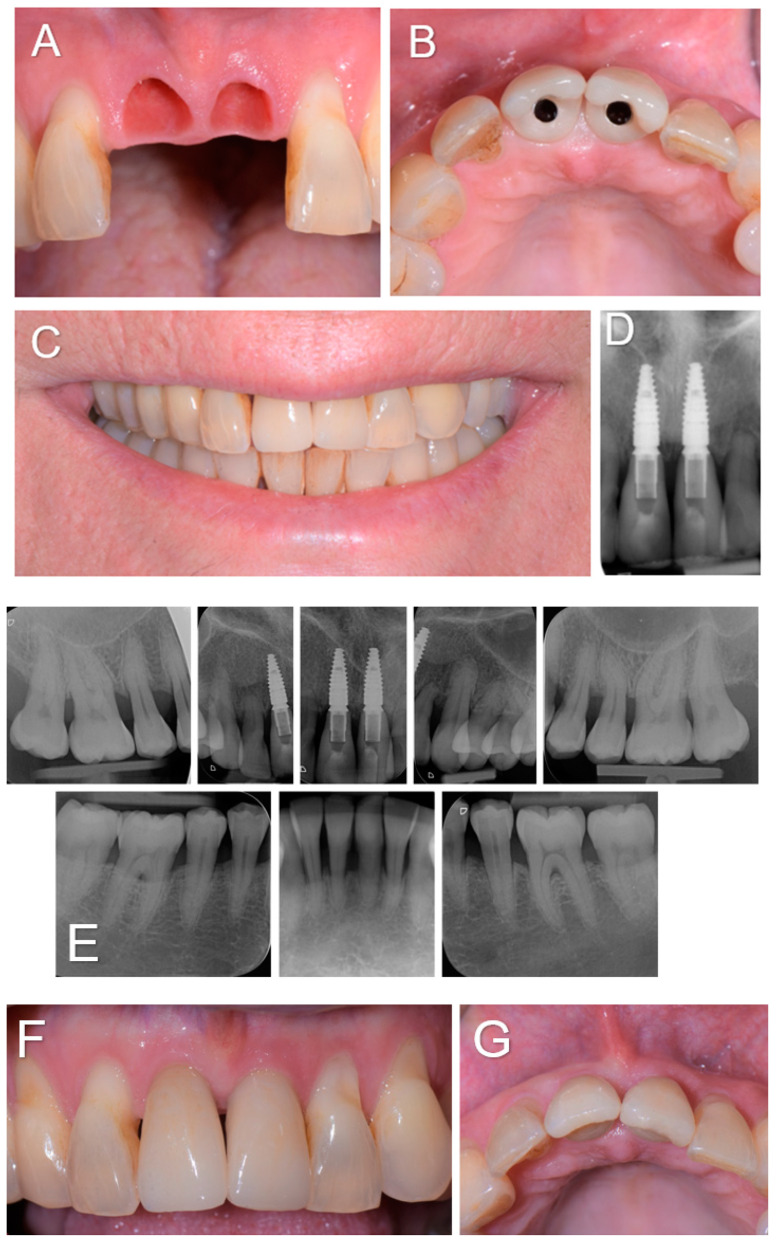
(**A**–**D**). Soft tissue condition when the two screw-retained single-unit crowns (E-max. press) were delivered 3 months post-operatively (**A**,**B**), and the clinical and radiographic examinations at the 3-year follow-up (**C**–**G**).

## Data Availability

The original data presented in the study are available on request from the corresponding author.
